# Supplementary material for: Gasdermin-A3 pore formation propagates along variable pathways
Source: Nat Commun. 2022 May 11;13:2609. doi: 10.1038/s41467-022-30232-8 (PMC9095878; doi:10.1038/s41467-022-30232-8)
Supplement: Supplementary file 1 — Supplementary Information [file 41467_2022_30232_MOESM1_ESM.pdf]

Supplementary Information

Gasdermin-A3 pore formation propagates along variable pathways

Stefania A. Mari[1,$], Kristyna Pluhackova[1,$,*], Joka Pipercevic[2], Matthew Leipner[1], Sebastian Hiller[2], Andreas Engel[1] & Daniel J. Müller[1,*]

[1] Department of Biosystems Science and Engineering, Eidgenössische Technische Hochschule (ETH) Zurich, 4058 Basel, Switzerland
[2] Biozentrum, University of Basel, 4056 Basel, Switzerland

[$]Contributed equally

*Corresponding authors: daniel.mueller@bsse.ethz.ch; kristyna.pluhackova@bsse.ethz.ch

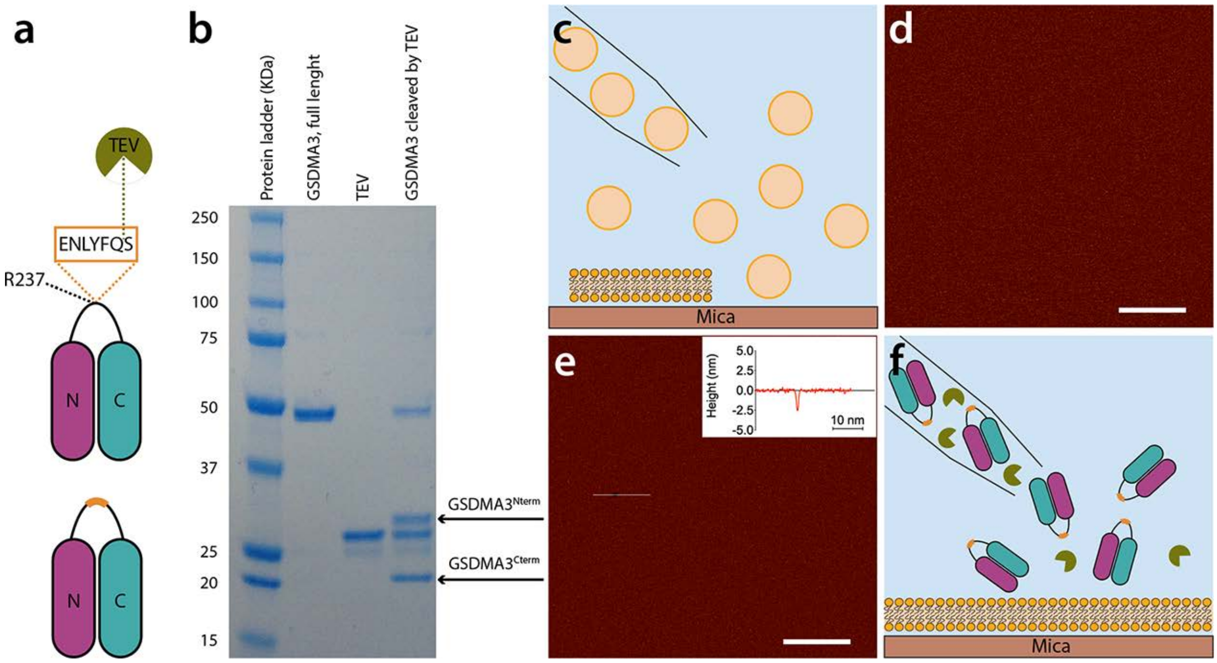

Supplementary Figure 1. Design of TEV-cleavable murine GSDMA3 (mGSDMA3) and AFM sample preparation. a, A tobacco etch virus protease (TEV) cleavage site was engineered directly following arginine 237 in the polypeptide loop connecting the N- and C-terminal domains of mGSDMA3. **b,** SDS-PAGE analysis of the enzymatic digestion of 1 µg mGSDMA3 by 1 µg TEV over night at 37°C. Cleavage of mGSDMA3 full-length (53 kDa) results in two fragments of 28 kDa (mGSDMA3^{Nterm}) and 25 kDa (mGSDMA3^{Cterm}). Molecular weight markers are annotated in kDa. Note that the TEV sample contains trace impurities, which do not affect the cleavage. **C,** For AFM, liposomes made from *E. coli* polar lipid extract are incubated on freshly cleaved mica in adsorption buffer solution (150 mM NaCl, 14 mM $CaCl_2$, 20 mM Hepes, pH 7.4) at room temperature. After an incubation time of ≈ 45 min, the support is gently rinsed with imaging buffer solution (50 mM NaCl, 0.1 mM TCEP, 0.1 mM EDTA, 20 mM Hepes, pH 7.4) to remove calcium and weakly attached liposomes and imaged by force-distance curve-based atomic force microscopy (FD-based AFM). **d,** FD-based AFM topograph of a defect-free supported lipid membrane (SLM). The SLM forms a continuous molecularly smooth surface so that its presence is hardly recognized in the topograph. **e,** The presence of the SLM becomes obvious in the very rare occasions when it has a defect (crossed by the thin white line in the FD-based AFM topograph). The height profile (red line) displayed in the insert shows the defect penetrating the membrane more clearly. The thickness of the supported lipid membrane corresponds to 5.0 ± 0.3 nm (mean ± SD, *n*=33). The full color range of both topographs corresponds to a vertical scale of 5 nm. Scale bars, 200 nm (**d**), 100 nm (**e**). Topographs were recorded in imaging buffer solution. **f,** After having confirmed the presence of a continuous and defect-free SLM by AFM imaging, a mGSDMA3^{Nterm} solution, which had been beforehand prepared by digesting full-length mGSDMA3 over night with TEV at 37°C, is incubated onto the SLM at 37°C for 3 h.

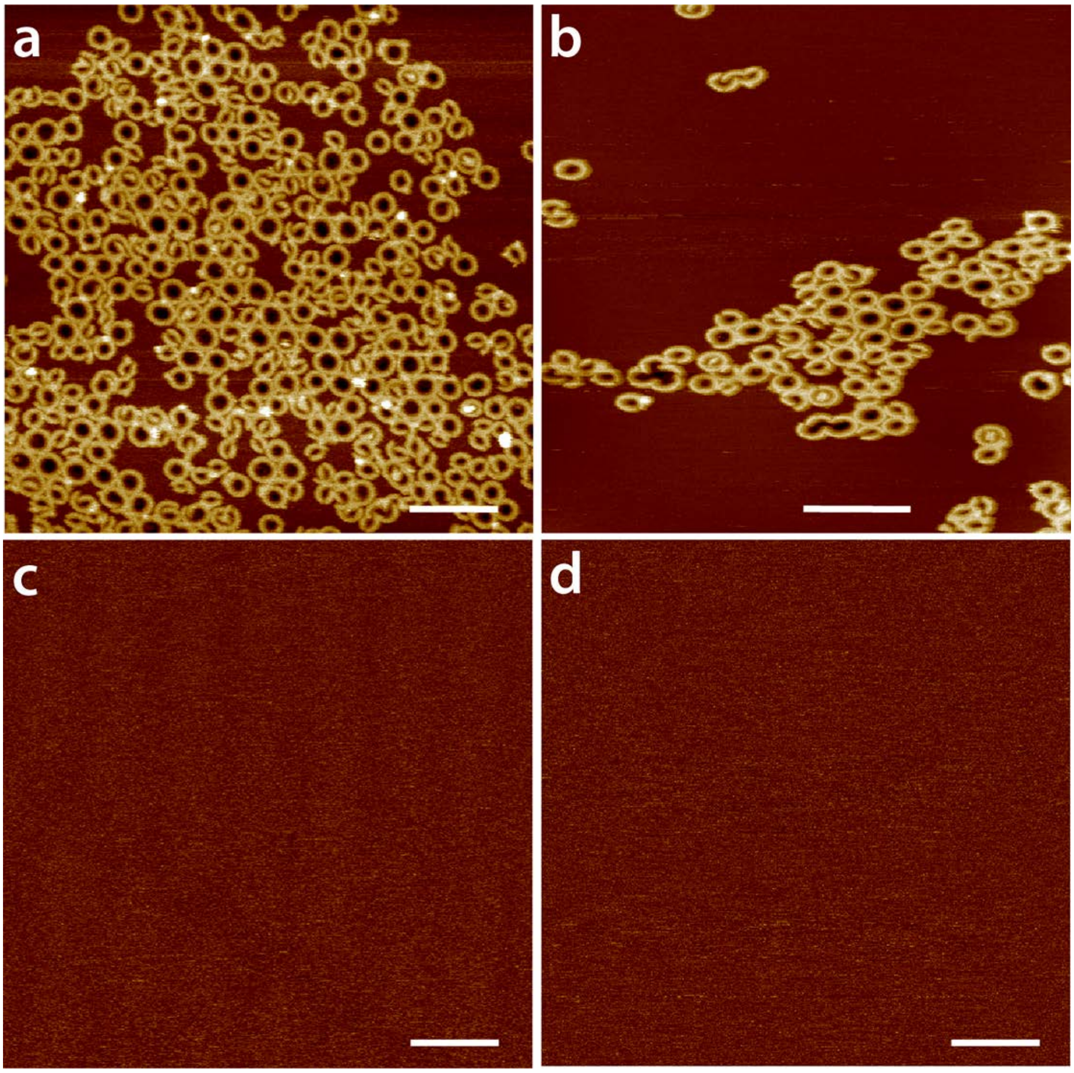

Supplementary Figure 2. mGSDMA3^{Nterm} oligomers insert and assemble into lipid membranes, whereas full-length mGSDMA3 or TEV do not. a, b, AFM topographs of mGSDMA3^{Nterm} oligomers formed on SLMs made from *E. coli* polar lipid extract. The topographs show mGSDMA3^{Nterm} oligomers inserted at different densities and complement the topographs shown in Fig. 1. **c, d**, Control experiments incubating SLMs of *E. coli* polar lipid extract with full-length mGSDMA3 or TEV. The AFM topographs show the SLM incubated with (**c**) 1.5 µM full-length mGSDMA3 and (**d**) 0.4 µM TEV. AFM topographs were recorded in imaging buffer solution at room temperature (Methods). The full-range color scale of the FD-based AFM topographs corresponds to a vertical scale of 8 nm (**a, b**) and 6 nm (**c, d**). Scale bars, 100 nm.

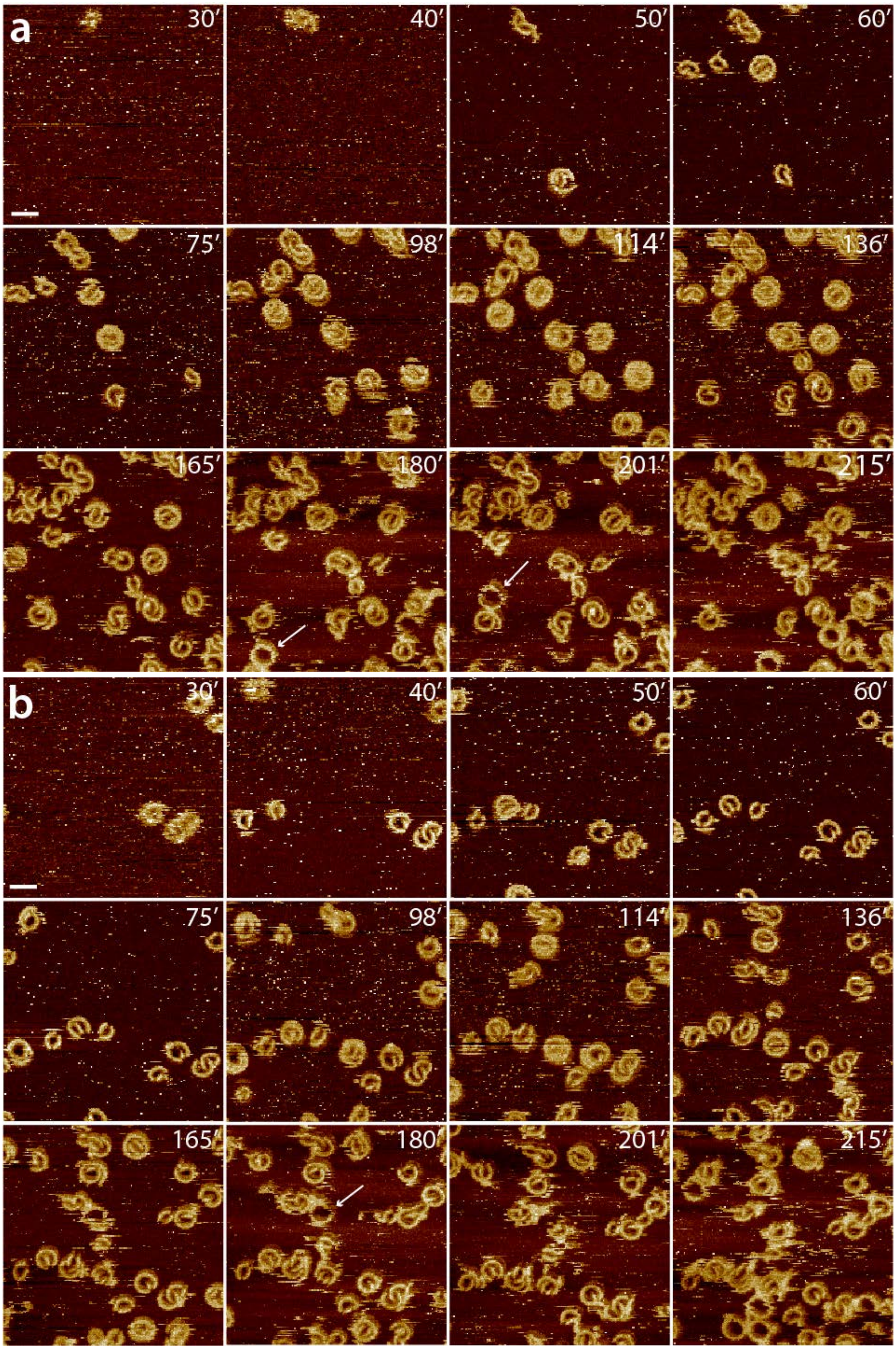

Supplementary Figure 3. Time-lapse AFM showing the assembly and growth of immobile membrane-inserted mGSDMA3^{Nterm} oligomers in the presence of mobile membrane-attached mGSDMA3^{Nterm} oligomers. Figure and legend are completed on next page.

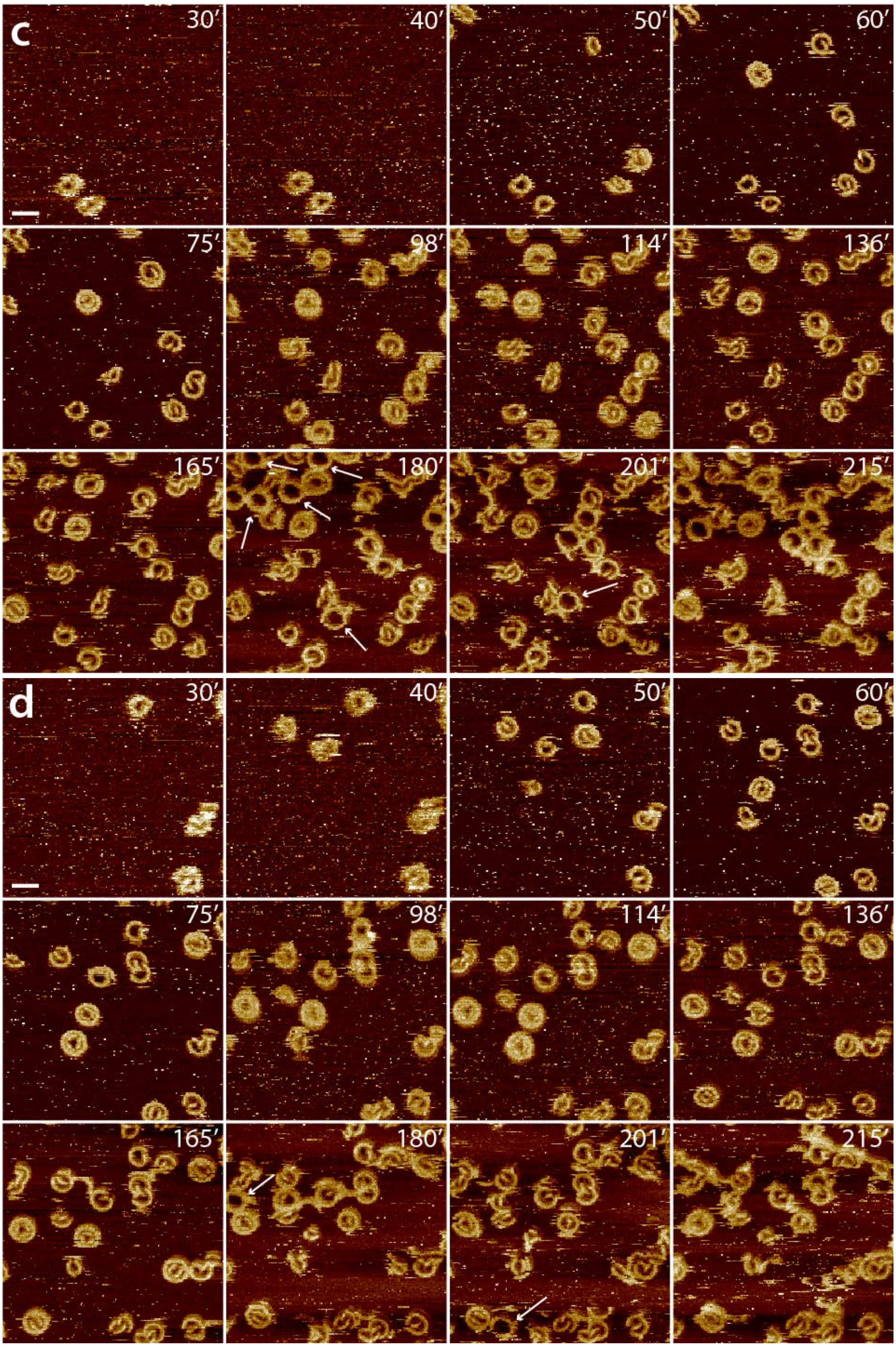

Supplementary Figure 3. Time-lapse AFM showing the assembly and growth of immobile membrane-inserted mGSDMA3^{Nterm} oligomers in the presence of mobile membrane-attached mGSDMA3^{Nterm} oligomers. **a–d,** A defect-free SLM made from *E. coli* polar lipid extract was incubated with a solution of 1.5 µM mGSDMA3, which had been beforehand cleaved with 0.4 µM TEV overnight at 37°C, and imaged by FD-based AFM in imaging buffer solution containing the cleaved mGSDMA3 at 37°C. Recorded at different time points of the incubation (time stamps indicate minutes) the time-lapse AFM topographs monitor mGSDMA3^{Nterm} insertion, assembly and growth into the SLM and also the assembly, disassembly and diffusion of mobile mGSDMA3^{Nterm} oligomers (white arrows). From **a** to **d**,

The time-lapse topographs show different areas of the SLM. The full-range color scale of the topographs corresponds to a vertical scale of 10 nm. Scale bars of 30 nm apply to all topographs. For further information on the representative time-lapse AFM series see Fig. 2 and Supplementary Movie 1. Please note that the mobile oligomers disappear by rinsing the sample with protein-free imaging buffer solution (Supplementary Fig. 4).

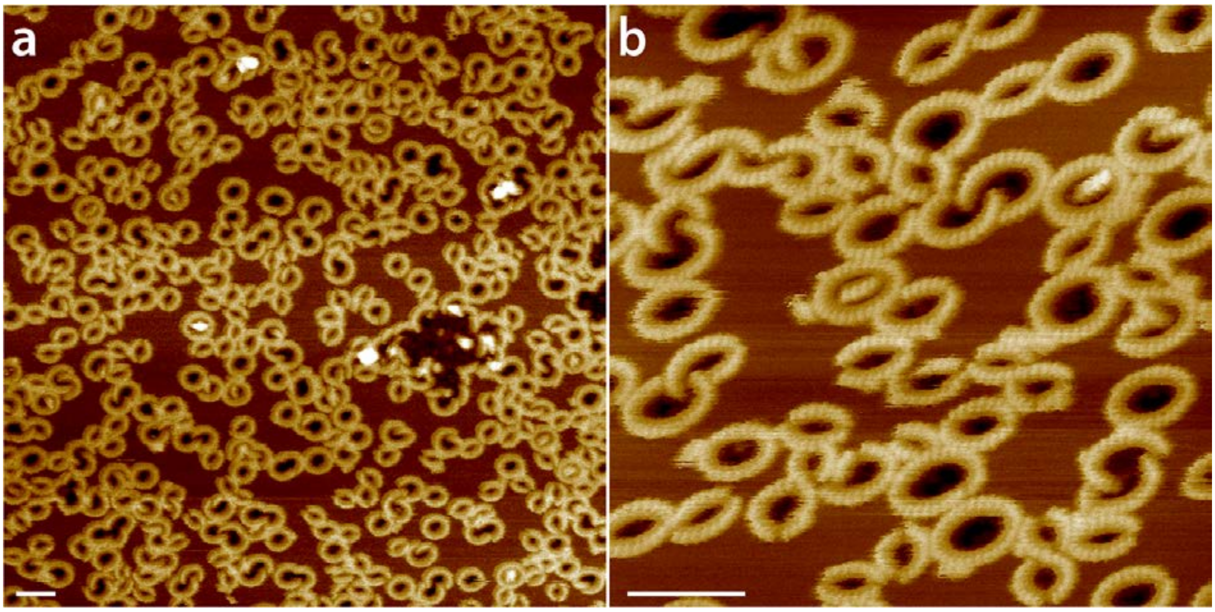

Supplementary Figure 4. AFM topographs of the SLM imaged in the time-lapse AFM experiment shown in Fig. 2 and Supplementary Fig. 3 but after rinsing to remove mGSDMA3 and TEV from the solution. a, b, AFM topographs recorded after rinsing the sample imaged in Fig. 2 and Supplementary Fig. 3 with imaging buffer solution containing no proteins. The FD-based AFM topographs show mGSDMA3^{Nterm} oligomers inserted into the SLM. The mobile oligomers, which were observed while incubating the SLM with mGSDMA3 and TEV (Fig. 2 and Supplementary Fig. 3) are not present anymore. Many oligomers of the immobile fraction show lytic transmembrane pores. The full-range color scales of the topographs, which were recorded in imaging buffer solution at room temperature, corresponds to vertical scales of 8.1 nm (**a**) and 9.7 nm (**b**). Scale bars, 40 nm.

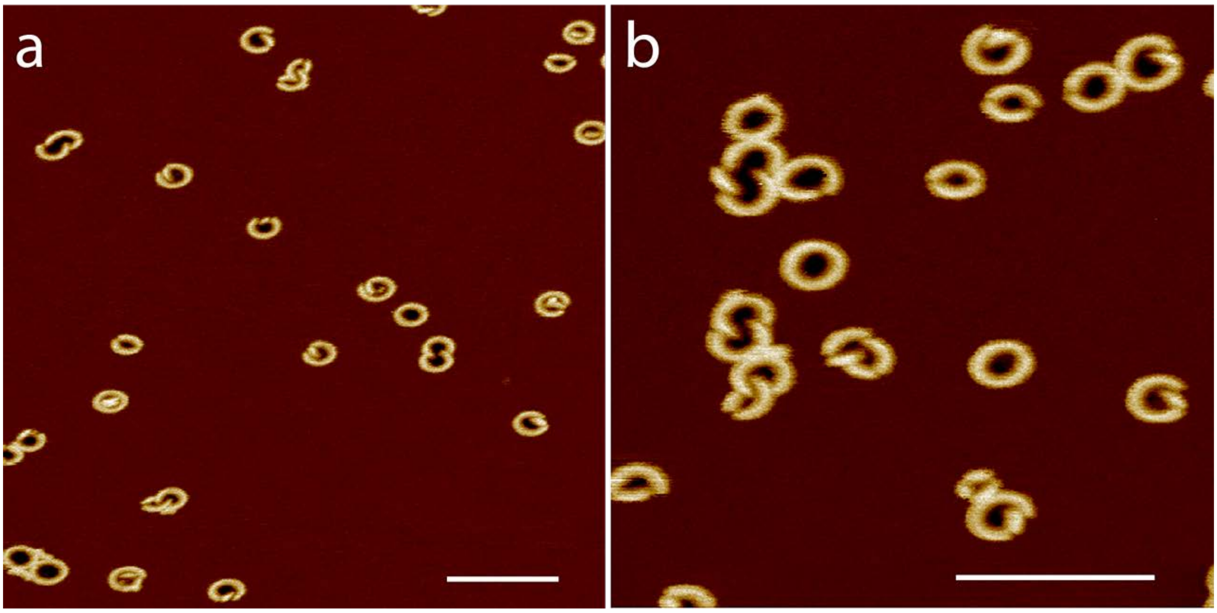

Supplementary Figure 5. AFM topographs showing the co-existence of arc-, slit- and ring-shaped mGSDMA3^{Nterm} oligomers several days after insertion in the SLM. a, b, The FD-based AFM topographs show the different oligomeric forms of mGSDMA3^{Nterm} to co-exist (**a**) seven and (**b**) nine days after their insertion in a lipid membrane. After insertion of mGSDMA3^{Nterm} into the SLM, the samples were kept in protein-free imaging buffer solution at 4°C. The full-range color scale of the topographs, which were recorded in imaging buffer solution, corresponds to a vertical scale of 5.1 nm. Scale bars, 100 nm.

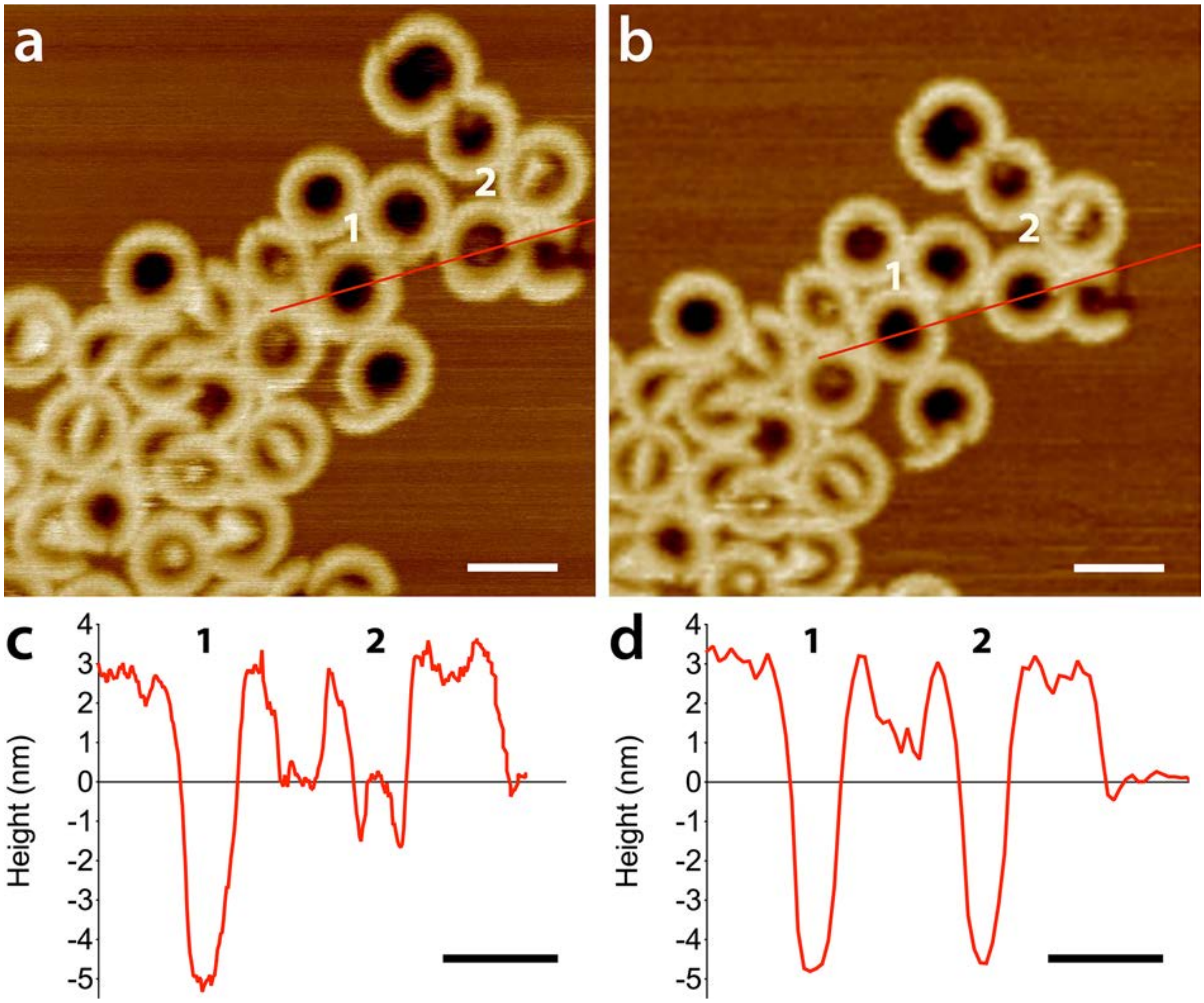

Supplementary Figure 6. Imaging a mGSDMA3^{Nterm} oligomer transiting from the pre-pore to the pore state in the absence of vertical collapse in supported lipid membranes made from *E. coli* polar lipid extract. a, b, FD-based AFM topographs recorded after each other show a mGSDMA3^{Nterm} oligomer transiting from the pre-pore (plugged) to a pore (unplugged) state. The red lines along the ring-shaped oligomers indicate the height profiles shown in **c** and **d**. Scale bars, 30 nm. The full-range color scale of the AFM topographs corresponds to a vertical scale of 10 nm. Imaging condition and buffer solution as described for the other AFM images (Methods). **c, d,** Height profiles showing the transition of mGSDMA3^{Nterm} oligomer no. 2 from the plugged, pre-pore state in **c** to the unplugged, pore state in **d**, whereas mGSDMA3^{Nterm} oligomer no. 1 remains in the unplugged, pore state. Independent of whether they reside in the pre-pore or pore state the oligomers show the same heights above the membrane. Scale bars, 30 nm.

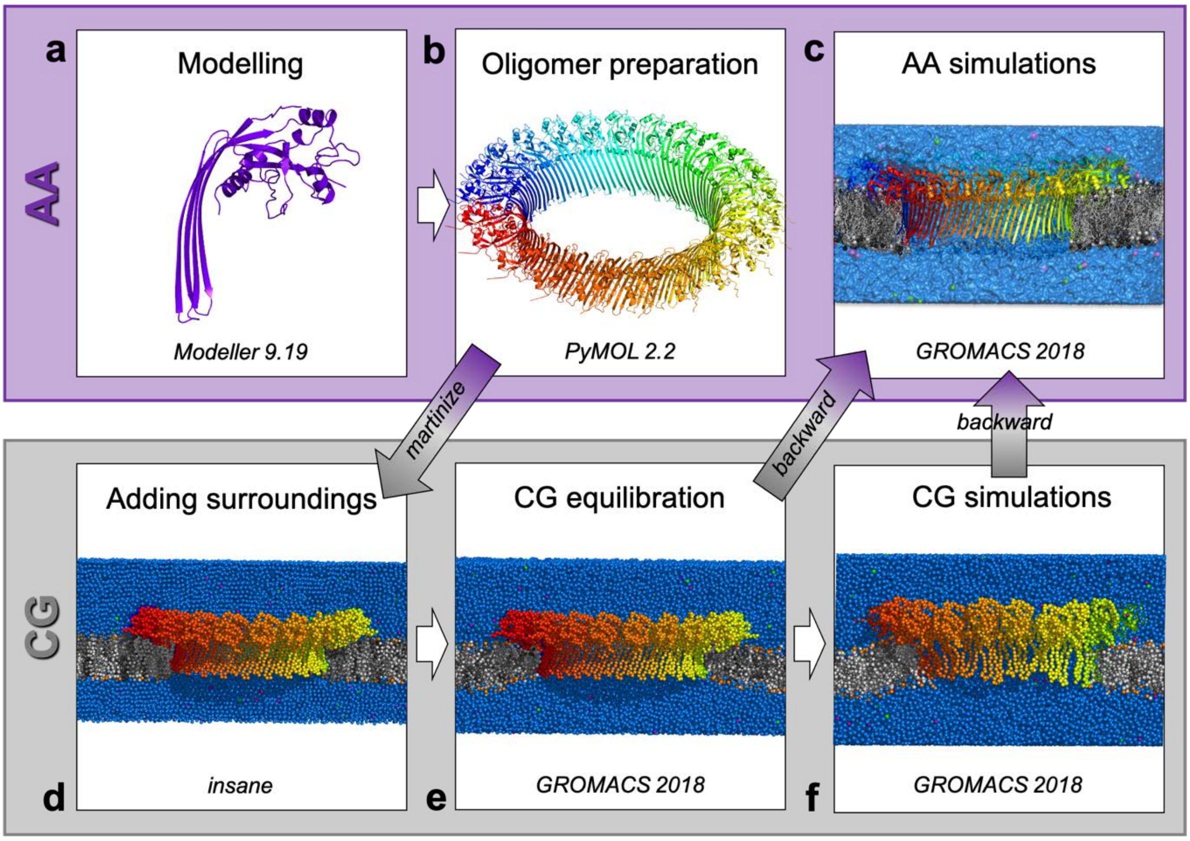

Supplementary Figure 7. Setup of sequential multiscaling molecular dynamics (MD) simulations. Simulation steps **a**, **b** and **c** (purple background) are performed at all-atom (AA) resolution and simulation steps **d**, **e**, and **f** (grey background) at coarse-grained (CG) resolution. **a**, Cryo-TEM structure of the mGSDMA3^{Nterm} subunit (PDB 6CB8)[1], which was completed by adding the residues 66-PGSS-69 and 234-KIRR-237 using Modeller 9.19[2] to build the structural model of mGSDMA3^{Nterm}. **b,** Using the atomistic model of the mGSDMA3^{Nterm} subunit (**a**), arc-, slit- and ring-shaped mGSDMA3^{Nterm} oligomers were prepared using PyMOL 2.4,[3] and coarse-grained to Martini3 force field[4] by the tool *martinize3* (kindly obtained from S.-J. Marrink). **c,** All-atom simulations were performed using the program GROMACS 2018.[5] **d,** After coarse-graining of the structures obtained in **b** to Martini3 force field[4] by the tool *martinize3* (kindly obtained from S.-J. Marrink), the lipid membrane of *E. coli* polar lipid extract[6], nonpolarizable water and 100 mM NaCl were added to the coarse-grained structure obtained in (**b**) by the tool *insane*[7]. **e,** During equilibration at coarse-grained resolution, the mGSDMA3^{Nterm} was position restrained. Coarse-grained MD simulations were performed using GROMACS 2018.[5] **f,** After simulation at coarse-grained resolution the system was converted to all-atom resolution using the tool *backward*[7].

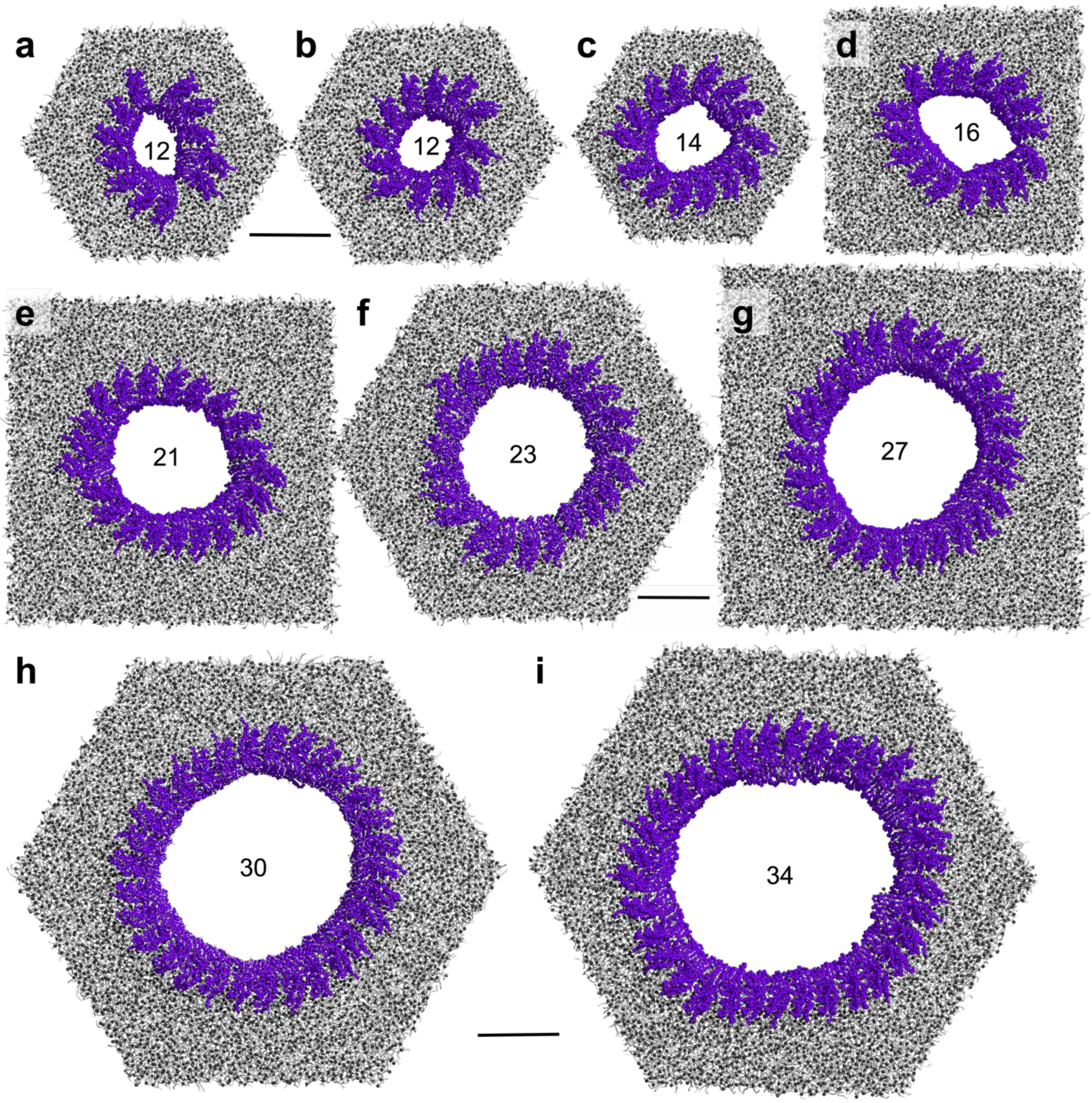

Supplementary Figure 8. Coarse-grained MD simulations of mGSDMA3^{Nterm} oligomers reshaping in membranes made from *E. coli* polar lipid extract. a-i, Reshaping of slit- and ring-shaped mGSDMA3^{Nterm} oligomers observed in coarse-grained MD simulations after an equilibration time of 4 µs. Before starting the simulations ring-shaped mGSDMA3^{Nterm} oligomers were assembled from 12 (**a, b**), 14 (**c**), 16 (**d**), 21 (**e**), 23 (**f**), 27 (g), 30 (**h**) or 34 (**i**) mGSDMA3^{Nterm}. mGSDMA3^{Nterm} are colored purple, lipid fatty acid chains and lipid headgroups are shown in light and dark grey, respectively. MD simulations were conducted at coarse-grained resolution. Scale bars, 10 nm.

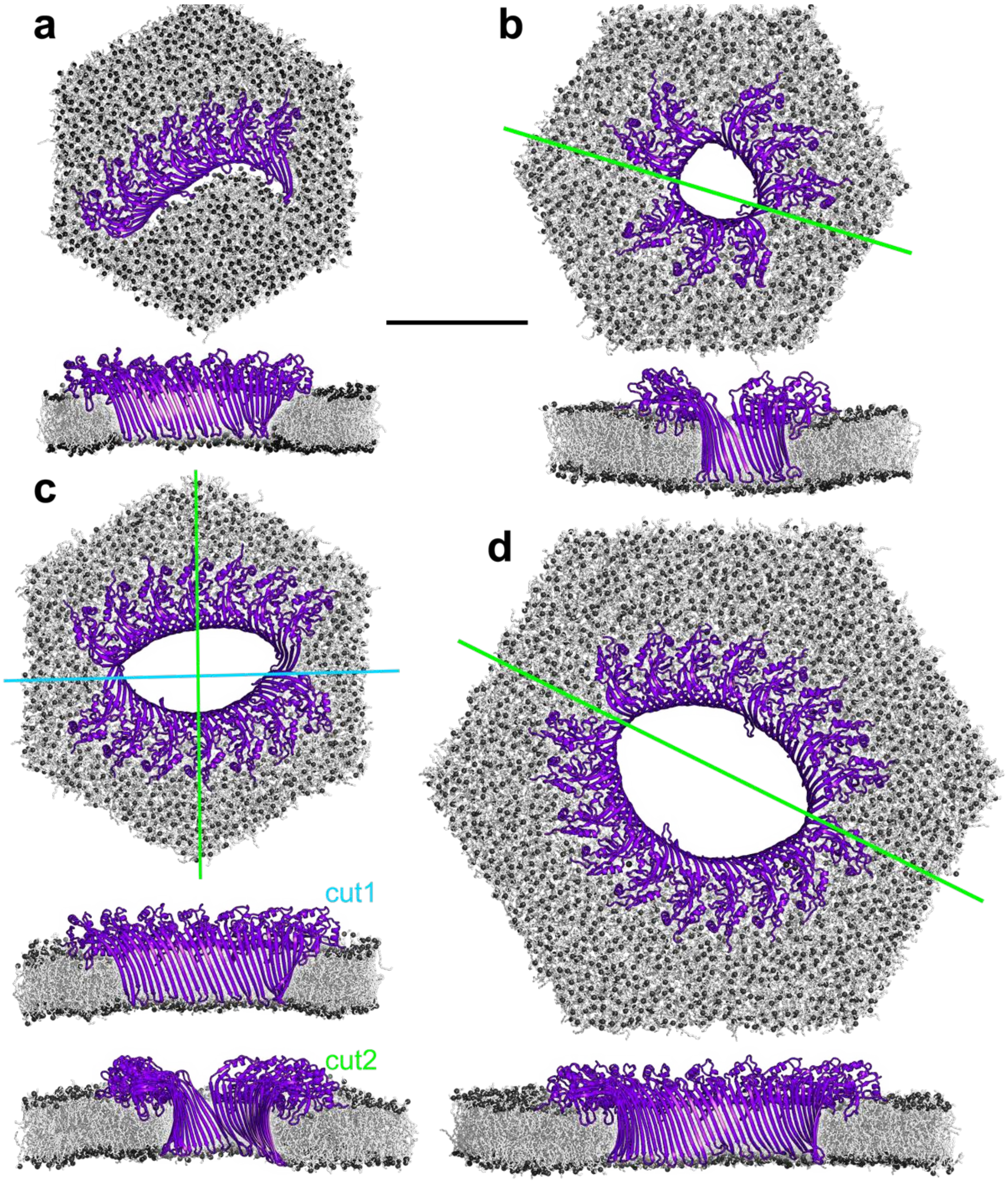

Supplementary Figure 9. All-atom MD simulations of different mGSDMA3^{Nterm} oligomers in membranes made from *E. coli* polar lipid extract. a, Top and sideview of an arc-shaped oligomer consisting of seven mGSDMA3^{Nterm} after 4 µs of simulation time. **b,** Top and cut-through side views (green line localizes the cut) of a ring-shaped oligomer, which reshaped into three arc-shaped oligomers within 1 µs of simulation time. Before starting the MD simulations, a ring-shaped oligomer was assembled from eight mGSDMA3^{Nterm}. **c,** Top and two cut-through side views of a slit-shaped oligomer after 1 µs of simulation time. Two different side views are highlighted by blue and green lines. Before starting the MD simulations two arc-shaped oligomers were assembled from seven mGSDMA3^{Nterm} each. **d,** Top and side views (green line localizes the side view) of a ring-shaped oligomer, which reshaped into a slit-shaped oligomer within 1.5 µs of simulation. Before starting the MD simulations, a ring-shaped oligomer was assembled from 18 mGSDMA3^{Nterm}. mGSDMA3^{Nterm} are colored purple, lipid fatty acid chains and lipid headgroups are shown in light and dark grey, respectively. MD simulations were conducted at all-atom resolution. Scale bar of 10 nm applies to all images.

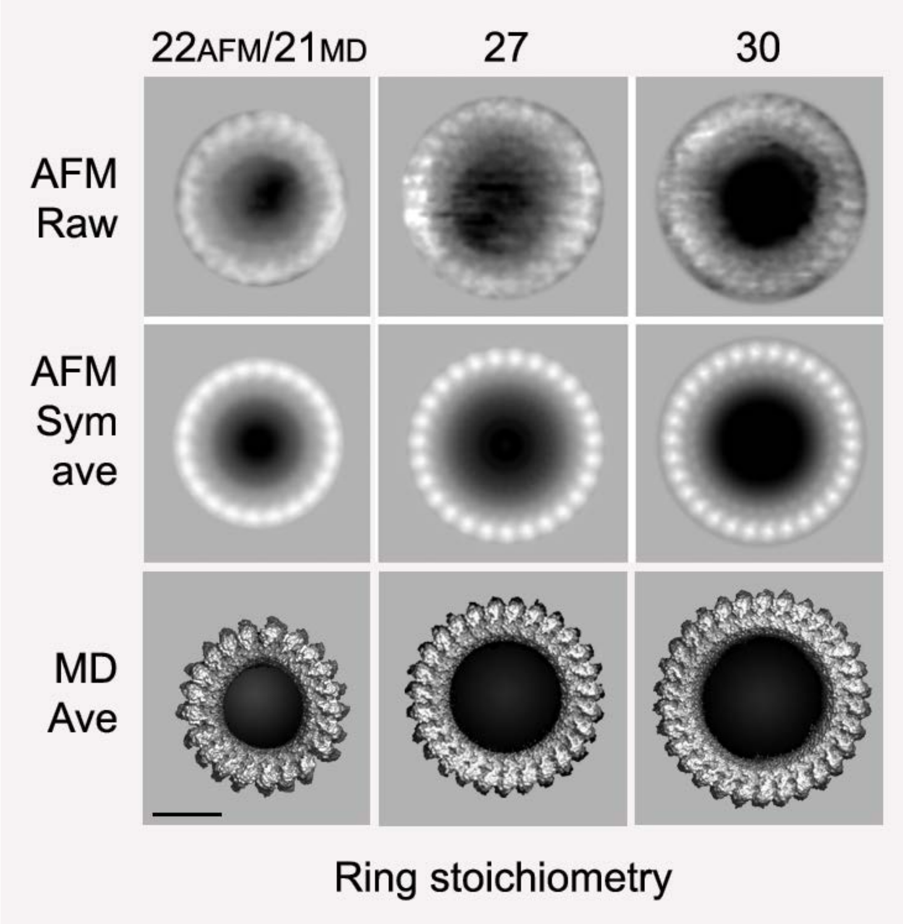

Supplementary Figure 10. Comparison of topographs of ring-shaped oligomers encompassing 22/21, 27 or 30 mGSDMA3^{Nterm} as obtained from MD simulations and AFM. Top row, selected high-resolution AFM topographs (raw data) of individual ring-shaped mGSDMA3^{Nterm} oligomers inserted into SLMs and having stoichiometries of 22, 27 and 30. Middle row, symmetrized correlation averages of mGSDMA3^{Nterm} oligomers imaged by AFM and showing stoichiometries of 22 (n = 11), 27 (n = 16) and 30 (n = 18) (Methods). n gives the number of oligomers averaged. Bottom row, topographs of ring-shaped oligomers of 21, 27 and 30 mGSDMA3^{Nterm} each derived from averaging 1’800 oligomers from two MD simulations. MD simulations were averaged from snapshots taken every ns, after exclusion of the first 100 ns of the simulation. Raw data and averages of AFM topographs were taken from Fig. 4. Scale bar of 10 nm applies to all images.

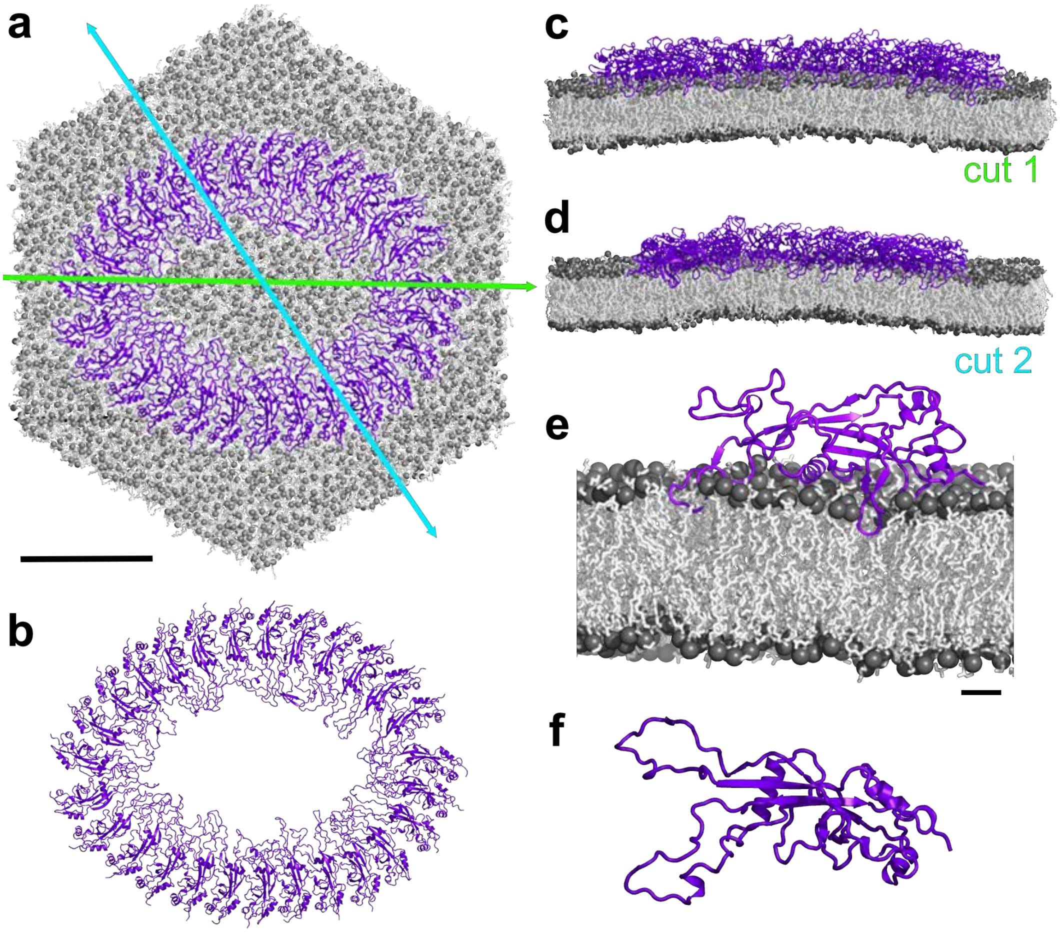

Supplementary Figure 11. Atomistic MD simulations of ring-shaped mGSDMA3^{Nterm} oligomers without transmembrane β-barrel adsorbed onto the lipid membrane. **a**, Membrane-attached ring-shaped oligomer of 27 mGSDMA3^{Nterm} subunits-reshaping within 500 ns into a slit. The oligomer was constructed by remodeling the β-hairpins of the cryo-TEM transmembrane oligomer[1] as polypeptide loops. While the head domains of ring-shaped mGSDMA3^{Nterm} oligomers without transmembrane β-barrel interact with the membrane surface in the same way as observed for membrane-inserted mGSDMA3^{Nterm}, the flexible and unstructured loops that would form β-hairpins in membrane-inserted oligomers only partially attach to the membrane surface. **b,** View of the mGSDMA3^{Nterm} oligomer from **a** without the membrane explicitly shown. **c,** Sideview on the mGSDMA3^{Nterm} oligomer after a cut along the green line in **a** (cut 1). **d,** Side view on the mGSDMA3^{Nterm} oligomer along the blue line in **a** (cut 2). **e,** Side view of one membrane adsorbed mGSDMA3^{Nterm} from **a**. **f,** Cytosolic view on a single membrane adsorbed mGSDMA3^{Nterm}, without the membrane explicitly shown. mGSDMA3^{Nterm} are shown in purple, lipids in grey. The scale bar of 10 nm in **a** applies also to **b**, **c**, and **d**. The scale bar of 1 nm in **a** also applies to **b** and **c**. The scale bar of 1 nm in **e** also applies to **f**.

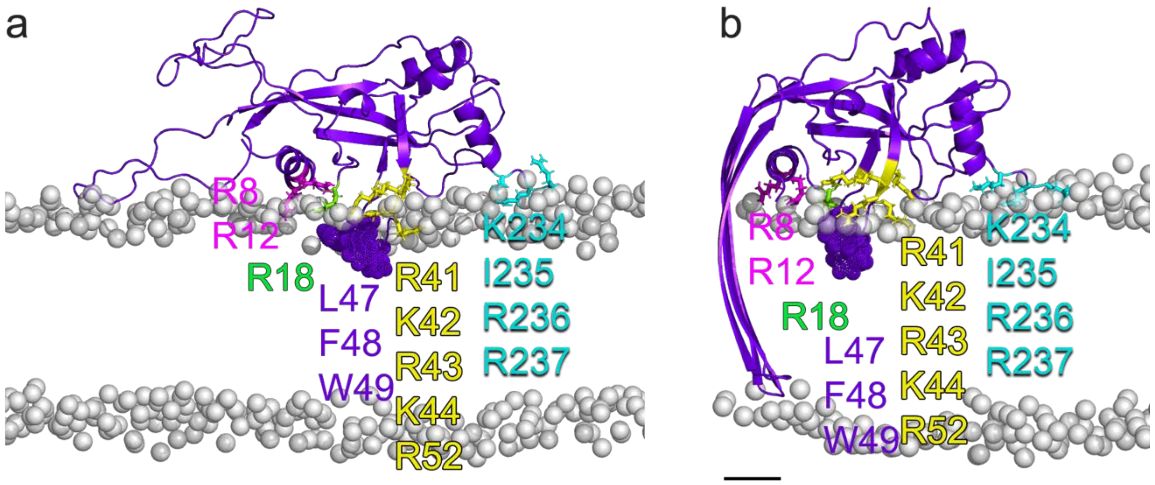

Supplementary Figure 12. Membrane contact sites of mGSDMA3^{Nterm} of (a) membrane-attached ring-shaped mGSDMA3^{Nterm} oligomers without β-barrel and (b) membrane-inserted ring-shaped mGSDMA3^{Nterm} oligomers with β-barrel. The contact sites, which appeared to be the same in both oligomers are exemplarily shown for one mGSDMA3^{Nterm} and include the positively charged α1 helix (residues R8 and R12 in magenta) and the β1–β2 loop, which features a hydrophobic tip (L47, F48, and W49 shown as purple mesh) flanked by basic residues R41, K42, R43, K44 and R52 (shown as yellow sticks). The simulations also reveal the positively charged, highly conserved R18, in the loop following the α1 helix, and the highly basic flexible C-terminus of mGSDMA3^{Nterm} (*i.e.*, residues K234, I235, R236 and R237, cyan sticks) to contact the negatively charged phosphates of the lipid headgroups (grey spheres). Shown are the representative contact sites of one mGSDMA3^{Nterm} from rings having (**a**) 27-fold and (**b**) 30-fold stoichiometries. Scale bar of 1 nm applies to both images.

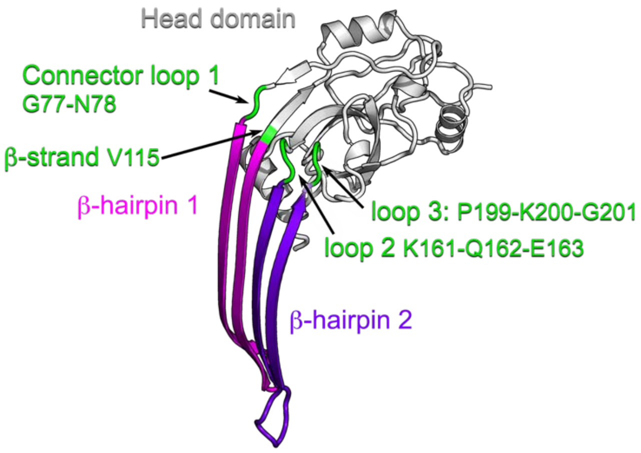

Supplementary Figure 13. Structure of the region (green) connecting both β-hairpin 1 (magenta) and 2 (purple) to the head domain (grey) of mGSDMA3^{Nterm}. While the β-strand residue V115 assures for the continuous connection of the β-strand of the transmembrane β-hairpin 1 and hydrophilic head domain, the three connector loops 1, 2, and 3 break the continuation of the remaining β-strands to the head domain. In detail, connector loop 1 contains a secondary structure breaking G (G77) and the connector loops 2 and 3 are comprised by "two-too-many" amino acids causing bulging of the connector loops and bestowing the structure a great flexibility. It is interesting to analyze the conservation of connector-forming residues within the gasdermin family: First, only 2 out of 16 available unique gasdermin sequences do not include a secondary structure breaking G or P residue in the connector loop 1. However, in the two G/P-lacking sequences (hGSDMB and mGSDME) additional amino acids precede the connector loop, thus giving a rise to a bulging loop, similar to the connector loops 2 and 3 described above for mGSDMA3. The connector β-strand residue V115 is highly conserved within the gasdermin family (11 out of 16 sequences include a V in this position, once I, once F, and three times K are alternatively present). The connector loop 2 of mGSDMA3 consists of the charged or polar residues K161-Q162-E163. In 10 out of 16 sequences a pair of charged amino acids (KE or KD) is found and the second or third position is always either a charged E or a polar Q or N. The connector loop 3, formed by P199-K200-G201 of mGSDMA3, is also very conserved in terms of secondary structure breaking P and G residues (14 out of 16 sequences contain P in the first position, and 12 out of 16 sequences contain G (and one P) in the third position.

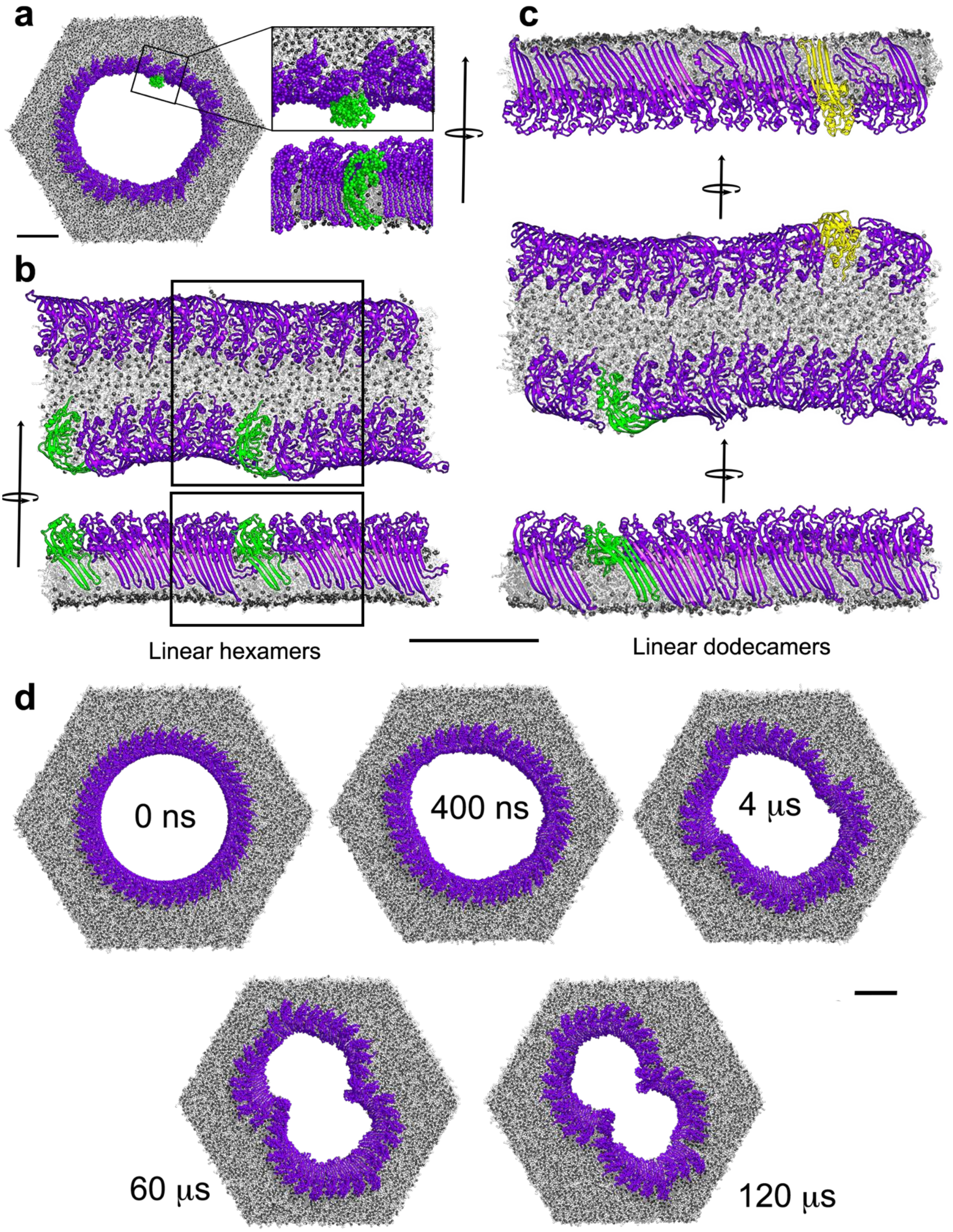

Supplementary Figure 14. MD simulations of membrane-inserted ring-shaped mGSDMA3^{Nterm} oligomers having too large radii. a, Reshaping of ring-shaped oligomers having 40 mGSDMA3^{Nterm}. mGSDMA3^{Nterm} are shown in purple, lipids in grey. After running coarse-grained simulations for 0.5 μs the contacts between β-hairpins of adjacent mGSDMA3^{Nterm} (green) disrupted, which was followed by the expulsion of the mGSDMA3^{Nterm} to the interior of the ring. See (**d**) for further development of the simulation. **b,** Defective hydrogen bonding network of mGSDMA3^{Nterm} oligomers assembled into linear periodic hexamers. The linear assembly of mGSDMA3^{Nterm} corresponds to a ring of infinite curvature.

After an equilibration time of 1 µs, the all-atom simulations reveal conformational changes similar to that observed in (**a**). The black frame highlights the position of the simulation box. The simulation system is visualized twice next to each other to highlight the contact of the linear oligomers over the periodic boundary conditions. **c,** Structural adaptation of mGSDMA3^{Nterm} oligomers linearly assembled into periodic dodecamers. After an all-atom simulation of 1 µs the hydrogen bonds between the β-sheets of some adjacent mGSDMA3^{Nterm} broke and the linear oligomer reshapes into smaller curved oligomers. mGSDMA3^{Nterm} partially expelled from the arc-shaped oligomers are highlighted in green and yellow. **d,** Reshaping of the ring-shaped oligomer assembled from 40 mGSDMA3^{Nterm}. Within hundreds of ns the ring-shaped oligomer started reshaping and within µs defects arose, which lead to the formation of smaller arc-shaped oligomers. Coarse-grained simulation times are indicated. Scale bars, 10 nm.

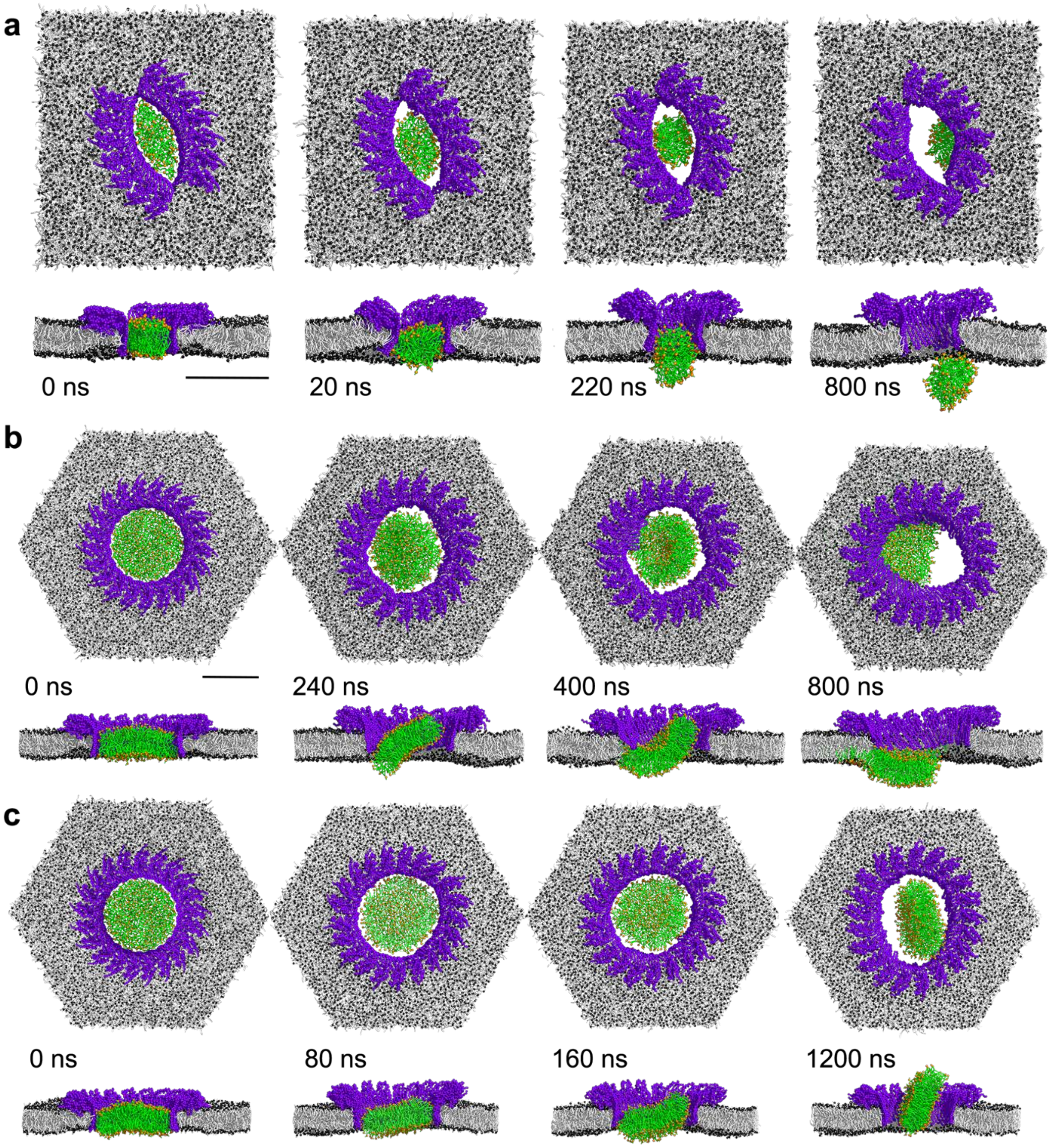

Supplementary Figure 15. Alternative pore forming pathways observed in coarse-grained MD simulations for different slit- and ring-shaped mGSDMA3^{Nterm} oligomers. **a,** Lipids leaving a slit-shaped oligomer as nanodisc (observed in 4 of 5 simulations, *n* = 4/5). The slit-shaped oligomer was formed by two arc-shaped oligomers each having seven mGSDMA3^{Nterm}. **b,** Lipids leaving a ring-shaped oligomer of 21 mGSDMA3^{Nterm} by fusing into the surrounding membrane (*n* = 3/9 simulations). **c,** Lipids leaving as a nanodisc a ring-shaped oligomer formed from 21 mGSDMA3^{Nterm} (*n* = 4/9 simulations). mGSDMA3^{Nterm} are colored purple, lipid fatty acid chains light grey and lipid headgroups dark grey. Lipids in the membrane pore are colored light green with orange headgroups. The top row in each subfigure shows the cytoplasmic view and the bottom row the side view. Scale bars, 10 nm. The figure complements Fig. 7.

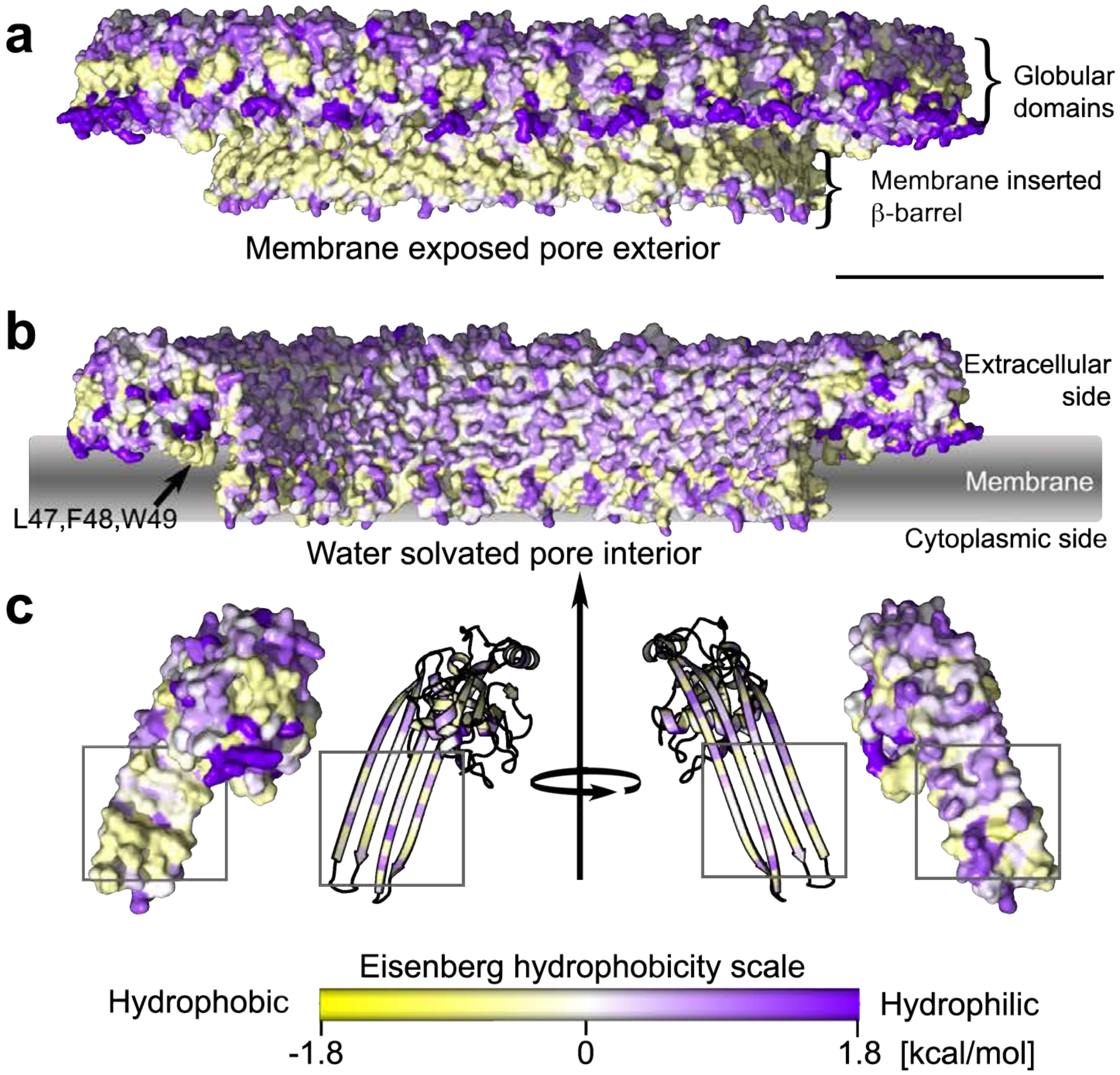

Supplementary Figure 16. Hydrophobicity of the surface of a membrane-inserted ring-shaped mGSDMA3^{Nterm} oligomer having 30-fold stoichiometry. The Eisenberg hydrophobicity scale of individual amino acids was calculated as described[8]. Hydrophobic neutral amino acids are colored yellow and hydrophilic residues purple. **a,** View along the outer surface of the mGSDMA3^{Nterm} oligomer with the membrane exposed pore exterior at the bottom and the water exposed globular head domains at the top. Dark purple residues at the boundary with the transmembrane β-barrel are positively charged amino acids that are adsorbed on the negatively charged membrane surface. **b,** Side view through the ring-shaped oligomer showing the water exposed inside of the transmembrane pore, which is covered by many polar residues providing a hydrophilic surface. Note the strongly hydrophobic loop Leu47, Phe48 and Trp49 in the globular head domain that reaches into the membrane and thus contributes to the stabilization of the hydrophilic head domain at the membrane surface. **c,** Views on the mGSDMA3^{Nterm} subunit from the membrane-exposed (left) and water-exposed (right) interface of the transmembrane pore. The cartoon representation shows an alternating pattern of hydrophobic (yellow) and hydrophilic (purple) residues, which is typical for transmembrane β-barrels[9]. Analysis of the hydrophobicity of the pore lining β-hairpins (grey squares) of mGSDMA3^{Nterm} based on the sum of hydrophobicities of individual amino acids[8] approximates a hydrophobicity of the membrane exposed surface (composed of 2 Ala, Asp, 4 Gly, 6 Leu, 1 Phe and 6 Val) of –7.53 kcal mol^{-1}. The water exposed (inner pore) surface of the β-hairpins (composed of Ala, 4 Asn, Asp, 3 Gln, Glu, 2 Gly, Ile, 2 Leu, 2 Lys, 2 Pro, 3 Ser and 3 Thr) shows a hydrophobicity of 7.27 kcal mol^{-1} (or hydrophilicity of –7.27 kcal mol^{-1}) with a clear preference for water. Scale bar, 10 nm.

Supplementary Table 1. Heights of membrane-inserted arc-, slit- and ring-shaped mGSDMA3^{Nterm} oligomers protruding from the lipid membrane and forming pre-pores or pores and membrane-attached ring-shaped oligomers. Pores are defined as oligomers forming a hole penetrating ≥ 2 nm into the lipid membrane. Heights were measured from AFM topographs. *n* gives the number of oligomers analyzed. Table correlates to Fig. 1i and 2j.

**Oligomeric shape**	**Pore/Pre-pore (depth ≥ 2 nm)**	***n***	**Height mean (nm)**	**Height SD (nm)**	**Height SE (nm)**
Arc	Membrane-inserted pre-pore	147	3.4	0.2	< 0.1
Arc	Membrane-inserted pore	161	3.4	0.2	< 0.1
Slit	Membrane-inserted pre-pore	127	3.5	0.3	< 0.1
Slit	Membrane-inserted pore	70	3.5	0.2	< 0.1
Ring	Membrane-inserted pre-pore	139	3.4	0.3	< 0.1
Ring	Membrane-inserted pore	77	3.4	0.3	< 0.1
Ring	Membrane-attached	203	3.4	0.3	< 0.1

Supplementary Table 2. List of performed MD simulations.

Simulated system	Resolution	Simulation time
Ring-shaped oligomer of 8 $mGSMDA3^{Nterm}$ with β-barrel	All-atom	1.5 µs, 1 µs
Ring-shaped oligomer of 18 $mGSMDA3^{Nterm}$ with β-barrel	All-atom	2 µs, 2 µs
Ring-shaped oligomer of 21 $mGSMDA3^{Nterm}$ with β-barrel	All-atom	1 µs, 1 µs
Ring-shaped oligomer of 27 $mGSMDA3^{Nterm}$ with β-barrel	All-atom	1 µs, 1 µs
Ring-shaped oligomer of 30 $mGSMDA3^{Nterm}$ with β-barrel	All-atom	1 µs, 1 µs
Arc-shaped oligomer of 7 $mGSMDA3^{Nterm}$ with β-barrel	All-atom	4 µs, 2.5 µs
Linear-shaped oligomer of two times 6 $mGSMDA3^{Nterm}$ with β-sheets	All-atom	1 µs
Linear-shaped oligomer of two times 12 $mGSMDA3^{Nterm}$ with β-sheets	All-atom	1 µs
Slit-shaped oligomer of two times 7 $mGSMDA3^{Nterm}$	All-atom	1 µs
Soluble ring-shaped oligomer of 27 $mGSMDA3^{Nterm}$ without β-barrel (β-hairpins remodeled as polypeptide loops) on membrane surface	All-atom	0.5 µs, 0.5 µs
Soluble ring-shaped oligomer of 27 $mGSMDA3^{Nterm}$ without β-barrel (β-hairpins remodeled as polypeptide loops) in solution	All-atom	0.1 µs
Ring-shaped oligomer of 12 $mGSMDA3^{Nterm}$ with β-barrel	Coarse-grained	4 x 4 µs
Ring-shaped oligomer of 21 $mGSMDA3^{Nterm}$ with β-barrel	Coarse-grained	9 x 4 µs
Ring-shaped oligomer of 23 $mGSMDA3^{Nterm}$ with β-barrel	Coarse-grained	5 x 4 µs
Ring-shaped oligomer of 27 $mGSMDA3^{Nterm}$ with β-barrel	Coarse-grained	13 x 4 µs
Ring-shaped oligomer of 30 $mGSMDA3^{Nterm}$ with β-barrel	Coarse-grained	10 x 4 µs
Ring-shaped oligomer of 34 $mGSMDA3^{Nterm}$ with β-barrel	Coarse-grained	5 x 4 µs
Ring-shaped oligomer of 40 $mGSMDA3^{Nterm}$ with β-barrel	Coarse-grained	120 µs, 60 µs
Slit-shaped oligomer of two times 7 $mGSMDA3^{Nterm}$ with β-sheets	Coarse-grained	2 x 4 µs
Arc-shaped oligomer of 7 $mGSMDA3^{Nterm}$ with β-sheets	Coarse-grained	0.8 µs, 0.4 µs
Arc-shaped oligomer of 16 $mGSMDA3^{Nterm}$ with β-sheets	Coarse-grained	0.8 µs, 2 µs, 2 µs, 2 µs
Arc-shaped oligomer of 20 $mGSMDA3^{Nterm}$ with β-sheets	Coarse-grained	0.8 µs, 0.4 µs, 0.4 µs, 0.4 µs
Slit-shaped oligomer of two times 7 $mGSMDA3^{Nterm}$ with β-sheets and lipids inside	Coarse-grained	0.8 µs, 0.8 µs, 2 µs, 2 µs, 4 µs
Ring-shaped oligomer of 21 $mGSMDA3^{Nterm}$ with β-barrel and lipids inside	Coarse-grained	0.8 µs, 0.8 µs, 0.8 µs, 1.2 µs, 0.4 µs, 0.8 µs, 0.8 µs
Ring-shaped oligomer of 27 $mGSMDA3^{Nterm}$ with β-barrel and lipids inside	Coarse-grained	2 x 4 µs, 4 x 40 µs, 2.7 µs
Ring-shaped oligomer of 30 $mGSMDA3^{Nterm}$ with β-barrel and lipids inside	Coarse-grained	6 x 2 µs

Supplementary References

1. Ruan, J., Xia, S., Liu, X., Lieberman, J. & Wu, H. Cryo-EM structure of the gasdermin A3 membrane pore. *Nature* **557**, 62-67 (2018).
2. Webb, B. & Sali, A. Comparative Protein Structure Modeling Using MODELLER. *Curr Protoc Bioinformatics* **54**, 5.6.1-5.6.37 (2016).
3. Schrödinger, L.L.C. The PyMOL Molecular Graphics System, Version 2.4 (2020).
4. Souza, P.C.T. et al. Martini 3: a general purpose force field for coarse-grained molecular dynamics. *Nat Methods* **18**, 382-388 (2021).
5. Abraham, M. et al. GROMACS: High performance molecular simulations through multi-level parallelism from laptops to supercomputers. *SoftwareX* **1-2**, 19-25 (2015).
6. Pluhackova, K. & Horner, A. CHARMM36 and Martini3 membrane models of E. coli polar lipid extract shed light on the importance of lipid composition complexity. *BMC Biology* (2020).
7. Wassenaar, T.A., Ingolfsson, H.I., Bockmann, R.A., Tieleman, D.P. & Marrink, S.J. Computational Lipidomics with insane: A Versatile Tool for Generating Custom Membranes for Molecular Simulations. *J Chem Theory Comput* **11**, 2144-2155 (2015).
8. Eisenberg, D., Weiss, R.M., Terwilliger, T.C. & Wilcox, W. Hydrophobic Moments and Protein-Structure. *Faraday Symp Chem S*, 109-120 (1982).
9. Wimley, W.C. Toward genomic identification of beta-barrel membrane proteins: composition and architecture of known structures. *Protein Sci* **11**, 301-312 (2002).
