# Supplementary material for: Gasdermin-A3 pore formation propagates along variable pathways
Source: Nat Commun. 2022 May 11;13:2609. doi: 10.1038/s41467-022-30232-8 (PMC9095878; doi:10.1038/s41467-022-30232-8)
Supplement: Supplementary file 3 — Description of Additional Supplementary Files [file 41467_2022_30232_MOESM3_ESM.pdf]

Supplementary Movie 1.
Representative time-lapse AFM experiment showing the assembly and growth of immobile membrane inserted mGSDMA3Nterm oligomers in the presence of mobile membrane attached mGSDMA3Nterm oligomers. The movie displays the entire sequence of time-lapse AFM topographs partially shown in Fig. 2 and Supplementary Fig. 3. The first FD-based AFM topograph shows a defect free SLM made from E. coli polar lipid extract. The defect-free SLM was incubated with a solution of 1.5 µM mGSDMA3, which had been beforehand cleaved with 0.4 µM TEV overnight at 37 °C, and imaged by FD-based AFM in imaging buffer solution containing the cleaved mGSDMA3 at 37 °C. Recorded at different time points of the incubation (time stamps indicate minutes) the time-lapse AFM topographs monitor mGSDMA3Nterm insertion, assembly, and growth into the SLM and also the assembly, disassembly and diffusion of mobile mGSDMA3Nterm oligomers. The full range color scale of the topographs corresponds to a vertical scale of 10 nm. Scale bar of 100 nm applies to all topographs. Please note that the mobile oligomers disappear by rinsing the sample with protein-free imaging buffer solution (Supplementary Fig. 4).

Supplementary Movie 2.
Pore formation of an arc-shaped oligomer assembled from 16 mGSDMA3Nterm and reshaping into a slit of 7 and 9 mGSDMA3Nterm . Cytoplasmic view. Coarse-grained MD simulation time 520 ns. mGSDMA3Nterm backbone atoms are shown as purple spheres, phosphates are shown as orange spheres. Lipids withdrawing from the transmembrane β-sheets thereby forming the transmembrane pore are colored green with white/orange/blue headgroups. The water is not shown for clarity.

Supplementary Movie 3.
Lipids leaving a ring-shaped oligomer of 21 mGSDMA3Nterm by fusing into the surrounding membrane. Cytoplasmic view. Coarse-grained MD simulation time 800 ns. mGSDMA3Nterm backbone atoms are shown as purple spheres, phosphates are shown as orange spheres. Lipids withdrawing from the transmembrane β-sheets thereby forming the transmembrane pore are colored green with white/orange/blue headgroups. The water is not shown for clarity.

Supplementary Movie 4.
Lipids leaving a ring-shaped oligomer of 21 mGSDMA3Nterm by fusing into the surrounding membrane. Side view. Coarse-grained MD simulation time 800 ns. mGSDMA3Nterm backbone atoms are shown as purple spheres, phosphates are shown as orange spheres. Lipids withdrawing from the transmembrane β-sheets thereby forming the transmembrane pore are colored green with white/orange/blue headgroups. The water is not shown for clarity.

Supplementary Movie 5.
Lipids leaving as a nanodisc a ring-shaped oligomer formed by 21 mGSDMA3Nterm . Cytoplasmic view. Coarse-grained MD simulation time 1’200 ns. mGSDMA3Nterm backbone atoms are shown as purple spheres, phosphates are shown as orange spheres. Lipids withdrawing from the transmembrane β-

sheets thereby forming the transmembrane pore are colored green with white/orange/blue headgroups. The water is not shown for clarity.

Supplementary Movie 6.
Lipids leaving as a nanodisc a ring-shaped oligomer formed by 21 mGSDMA3Nterm . Side view. Coarse grained MD simulation time 1’200 ns. mGSDMA3Nterm backbone atoms are shown as purple spheres, phosphates are shown as orange spheres. Lipids withdrawing from the transmembrane β-sheets thereby forming the transmembrane pore are colored green with white/orange/blue headgroups. The water is not shown for clarity.

Supplementary Movie 7.
Lipids leaving as a vesicle a ring-shaped oligomer consisting of 30 mGSDMA3Nterm . Cytoplasmic view. Coarse-grained MD simulation time 260 ns. mGSDMA3Nterm backbone atoms are shown as purple spheres, phosphates are shown as orange spheres. Lipids withdrawing from the transmembrane β-sheets thereby forming the transmembrane pore are colored green, with white/orange/blue headgroups. The water is not shown for clarity.

Supplementary Movie 8.
Lipids leaving as a vesicle a ring-shaped oligomer consisting of 30 mGSDMA3Nterm . Side view. Coarse grained MD simulation time 260 ns. mGSDMA3Nterm backbone atoms are shown as purple spheres, phosphates are shown as orange spheres. Lipids withdrawing from the transmembrane β-sheets thereby forming the transmembrane pore are colored green with white/orange/blue headgroups. The water is not shown for clarity.

Supplementary Movie 9.
Lipids leaving a symmetric slit-shaped oligomer assembled from 14 mGSDMA3Nterm by fusing into the surrounding membrane. Cytoplasmic view. Coarse-grained MD simulation time 1’600 ns. mGSDMA3Nterm backbone atoms are shown as purple spheres, phosphates are shown as orange spheres. Lipids withdrawing from the transmembrane β-sheets thereby forming the transmembrane pore are colored green with white/orange/blue headgroups. The water is not shown for clarity.

Supplementary Movie 10.
Lipids leaving a symmetric slit-shaped oligomer assembled from 14 mGSDMA3Nterm by fusing into the surrounding membrane. Side view. Coarse-grained MD simulation time 1’600 ns. mGSDMA3Nterm backbone atoms are shown as purple spheres, phosphates are shown as orange spheres. Lipids withdrawing from the transmembrane β-sheets thereby forming the transmembrane pore are colored green with white/orange/blue headgroups. The water is not shown for clarity.
