# Supplementary material for: Gasdermin-A3 pore formation propagates along variable pathways
Source: Nat Commun. 2022 May 11;13:2609. doi: 10.1038/s41467-022-30232-8 (PMC9095878; doi:10.1038/s41467-022-30232-8)
Supplement: Supplementary file 14 — Reporting Summary [file 41467_2022_30232_MOESM14_ESM.pdf]

nature research

Corresponding author(s): Daniel J. Müller, Kristyna Pluhackova

Last updated by author(s): Apr 2, 2022

Reporting Summary

Nature Research wishes to improve the reproducibility of the work that we publish. This form provides structure for consistency and transparency in reporting. For further information on Nature Research policies, see our Editorial Policies and the Editorial Policy Checklist.

Statistics

For all statistical analyses, confirm that the following items are present in the figure legend, table legend, main text, or Methods section.

n/a	Confirmed	
[ ]	[x]	The exact sample size (n) for each experimental group/condition, given as a discrete number and unit of measurement
[ ]	[x]	A statement on whether measurements were taken from distinct samples or whether the same sample was measured repeatedly
[x]	[ ]	The statistical test(s) used AND whether they are one- or two-sided<br>*Only common tests should be described solely by name; describe more complex techniques in the Methods section.*
[x]	[ ]	A description of all covariates tested
[x]	[ ]	A description of any assumptions or corrections, such as tests of normality and adjustment for multiple comparisons
[ ]	[x]	A full description of the statistical parameters including central tendency (e.g. means) or other basic estimates (e.g. regression coefficient) AND variation (e.g. standard deviation) or associated estimates of uncertainty (e.g. confidence intervals)
[x]	[ ]	For null hypothesis testing, the test statistic (e.g. F, t, r) with confidence intervals, effect sizes, degrees of freedom and P value noted<br>*Give P values as exact values whenever suitable.*
[x]	[ ]	For Bayesian analysis, information on the choice of priors and Markov chain Monte Carlo settings
[x]	[ ]	For hierarchical and complex designs, identification of the appropriate level for tests and full reporting of outcomes
[x]	[ ]	Estimates of effect sizes (e.g. Cohen's d, Pearson's r), indicating how they were calculated

Our web collection on statistics for biologists contains articles on many of the points above.

Software and code

Policy information about availability of computer code

Data collection	For recording of the AFM data we used the software (NanoScope Analysis 1.8) provided by the AFM manufacturer (Bruker/Veeco Instruments). To conduct MD simulations we used the software GROMACS 2018.
Data analysis	For AFM data analysis (height and diameter analysis of oligomers) we used the software (NanoScope Analysis 1.8) provided by the AFM manufacturer (Bruker/Veeco Instruments). For correlation averaging of the high resolution AFM Data we used the Semper image processing software. For data analysis and processing of the MD simulations we used the softwares python 3, R 4, GROMACS 2018, modeller 9.19, and PyMOL 2.4. Please note that LINCS and CHARMM36m are no software packages on their own. LINCS is included in GROMACS and CHARMM36m is the force field, which we used for analysis in the absence of the CHARMM programm.

For manuscripts utilizing custom algorithms or software that are central to the research but not yet described in published literature, software must be made available to editors and reviewers. We strongly encourage code deposition in a community repository (e.g. GitHub). See the Nature Research guidelines for submitting code & software for further information.

Data

Policy information about availability of data

All manuscripts must include a data availability statement. This statement should provide the following information, where applicable:
- Accession codes, unique identifiers, or web links for publicly available datasets
- A list of figures that have associated raw data
- A description of any restrictions on data availability

All data and codes are available upon resonable request

Field-specific reporting

Please select the one below that is the best fit for your research. If you are not sure, read the appropriate sections before making your selection.

- [x] Life sciences
- [ ] Behavioural & social sciences
- [ ] Ecological, evolutionary & environmental sciences

For a reference copy of the document with all sections, see nature.com/documents/nr-reporting-summary-flat.pdf

Life sciences study design

All studies must disclose on these points even when the disclosure is negative.

Sample size	The sample size of molecular dynamics (MD) simulations was limited by computational resources. We have performed every simulation at least in two replicas for each oligomeric shape and size. The properties analyzed in our atomistic simulations resulted from sample sizes ranging from 180000–680000 data points for two 0.1–2 µs simulations of rings containing 8 GASDMA3 Nterm and two 0.1–1 µs simulations of 40 GASDMA3 Nterm. The full data is shown as box plots throughout the manuscript showing the spread of the data. The rationale for the sample sizes was done by comparing average chain properties (e.g. number of hydrogen bonds) between the two simulation replicas. For example in the two simulations containing 27 GASDMA3Nterm protomers the p-values between the number of hydrogen bonds formed between the beta-hairpins or between the globular headgroups amounted to p=0.9972 and 0.8939 indicating that the results from the two simulations are statistically similar and thus the sample size is sufficient.
Data exclusions	From MD simulations we excluded the first 100 ns of the simulations from analysis of all-atom simulations for equilibration purposes. The exclusion of the first 100 ns was pre-established in the field and is based on the experience that the protein and membrane need tens of ns to adapt, which is indicated by a steep change of the root mean square deviation of the simulated protein structure from the cryo-EM structure. Overall each MD simulation lastet at least 1000 ns. No data exclusion was done in case of coarse-grained simulations. No data exclusion was applied for the analysis of the AFM data.
Replication	Each all-atom simulation of oligomeric shapes was performed at least twice, the list of all simulations, their length and number of replicas is included as Supplementary Table S3. Every AFM experiment was repeated at least three times with each experiment characterizing independent and new sample preparations, new AFM supports, and new AFM cantilevers. In some cases the AFM experiments have been repeated more than 20 times. Each AFM experiment conducted under certain experimental condition provided reproducible results for the experimental condition. Occassionally the AFM experiments had to be repeated because the prepartion of a defect free supported lipid membrane (SLM) did not work properly, the AFM fluid cell was contaminated, or the AFM tip was contaminated.
Randomization	AFM and TEM experiments characterizing different sample conditions were done in random order and repeated over the time course of several weeks. The experimental conditions applied for each AFM and TEM experiment are defined in the manuscript. Every AFM and TEM experiment conducted under a given experimental condition has been allocated to the same group of condition.
Blinding	Blinding was not relevant to this study as the experimental or simulations conditions had to be defined from the beginning of each experiments or simulation. Each experimental and simulation condition revealed reproducible and distinct results.

Reporting for specific materials, systems and methods

We require information from authors about some types of materials, experimental systems and methods used in many studies. Here, indicate whether each material, system or method listed is relevant to your study. If you are not sure if a list item applies to your research, read the appropriate section before selecting a response.

Materials & experimental systems

n/a	Involved in the study	
x		Antibodies
x		Eukaryotic cell lines
x		Palaeontology and archaeology
x		Animals and other organisms
x		Human research participants
x		Clinical data
x		Dual use research of concern

Methods

n/a	Involved in the study	
x		ChIP-seq
x		Flow cytometry
x		MRI-based neuroimaging
